# Lifetime study in mice after acute low-dose ionizing radiation: a multifactorial study with special focus on cataract risk

**DOI:** 10.1007/s00411-017-0728-z

**Published:** 2018-01-11

**Authors:** Claudia Dalke, Frauke Neff, Savneet Kaur Bains, Scott Bright, Deborah Lord, Peter Reitmeir, Ute Rößler, Daniel Samaga, Kristian Unger, Herbert Braselmann, Florian Wagner, Matthias Greiter, Maria Gomolka, Sabine Hornhardt, Sarah Kunze, Stefan J. Kempf, Lillian Garrett, Sabine M. Hölter, Wolfgang Wurst, Michael Rosemann, Omid Azimzadeh, Soile Tapio, Michaela Aubele, Fabian Theis, Christoph Hoeschen, Predrag Slijepcevic, Munira Kadhim, Michael Atkinson, Horst Zitzelsberger, Ulrike Kulka, Jochen Graw

**Affiliations:** 1 Helmholtz Center Munich, German Research Center for Environmental Health, Institute of Developmental Genetics, 85764 Neuherberg, Germany; 2 Helmholtz Center Munich, German Research Center for Environmental Health, Institute of Pathology, Neuherberg, Germany; 3 Department of Life Sciences, Brunel University London, Uxbridge, UK; 4 Department of Biological and Medical Sciences, Oxford Brookes University, Oxford, UK; 5 Helmholtz Center Munich, German Research Center for Environmental Health, Institute of Health Economics and Health Care Management, Neuherberg, Germany; 6 Department Radiation Protection and Health, Federal Office for Radiation Protection, Oberschleissheim, Germany; 7 Helmholtz Center Munich, German Research Center for Environmental Health, Research Unit of Radiation Cytogenetics, Neuherberg, Germany; 8 Helmholtz Center Munich, German Research Center for Environmental Health, Research Unit Medical Radiation Physics and Diagnostics, Neuherberg, Germany; 9 Helmholtz Center Munich, German Research Center for Environmental Health, Institute of Radiation Biology, Neuherberg, Germany; 10 Helmholtz Center Munich, German Research Center for Environmental Health, Institute of Computational Biology, Neuherberg, Germany; 11 Present Address: University of Texas, MD Anderson, Houston, TX USA; 12 Present Address: Helmholtz Center Munich, German Research Center for Environmental Health, Individual Monitoring Service, Neuherberg, Germany; 13 Present Address: Chair of Medical Systems Technology, Otto-von-Guericke University Magdeburg, Magdeburg, Germany; 14 Present Address: Department of Biological and Medical Sciences, Oxford Brookes University, Oxford, UK; 15 Present Address: Department of Bioanalytical Sciences, CSL Behring GmbH, Marburg, Germany; 16 Present Address: Municipal Clinical Center Munich, Munich, Germany; 17 Present Address: Helmholtz Center Munich, German Research Center for Environmental Health, Institute of Radiation Protection, Neuherberg, Germany; 18 Present Address: Helmholtz Center Munich, German Research Center for Environmental Health, Research Unit of Radiation Cytogenetics, Neuherberg, Germany

**Keywords:** Radiation-induced cataract, Mouse, Low-dose radiation, Scheimpflug analysis

## Abstract

Because of the increasing application of ionizing radiation in medicine, quantitative data on effects of low-dose radiation are needed to optimize radiation protection, particularly with respect to cataract development. Using mice as mammalian animal model, we applied a single dose of 0, 0.063, 0.125 and 0.5 Gy at 10 weeks of age, determined lens opacities for up to 2 years and compared it with overall survival, cytogenetic alterations and cancer development. The highest dose was significantly associated with increased body weight and reduced survival rate. Chromosomal aberrations in bone marrow cells showed a dose-dependent increase 12 months after irradiation. Pathological screening indicated a dose-dependent risk for several types of tumors. Scheimpflug imaging of the lens revealed a significant dose-dependent effect of 1% of lens opacity. Comparison of different biological end points demonstrated long-term effects of low-dose irradiation for several biological end points.

**Electronic supplementary material:**

The online version of this article (10.1007/s00411-017-0728-z) contains supplementary material, which is available to authorized users.

## Introduction

For decades, it is well known that high doses of X-rays cause cataracts (i.e., opacities of the ocular lens). Several epidemiological studies indicate that the threshold for cataract development is less than 2 Gy, possibly in the range of 0.5 Gy (for reviews see Ainsbury et al. 2009, 2016). A more recent study by Azizova et al. (2016) showed in a cohort of Mayak workers the highest relative risk for cataracts at doses above 2.0 Gy. In mice, the most recent studies indicated that radiation-induced and vision-impairing cataracts can clearly be detected 1 year after irradiation by 0.5 Gy (X-ray) in 50% of wild-type mice and to a much higher extent in heterozygous *Atm* or *Mrad9* mutant mice (Kleiman et al. 2007).

Because of the ongoing discussion concerning the dose limit or threshold for cataract formation after low-dose exposure, we designed a new lifetime experiment in mice using the dose of 0.5 Gy as a positive control (according to Kleiman et al. 2007) and additional doses of 0.125 and 0.063 Gy as low doses. Since we did whole-body irradiation without shielding the mice, radiation-induced damages are not restricted to the germinative zone of the lens, but may affect also other regions of the lens, the eye and the entire body.

To detect any type of cataract, we used the Scheimpflug technique, which was demonstrated to be more precise and objective than lens opacity classification systems: the Scheimpflug technique relies on densitometric features from the anterior corneal surface to the posterior lens surface instead of morphological features as used by classical scoring systems (Wegener and Laser-Junga 2009). In mice, this system was introduced into the routine work of the German Mouse Clinic (Fuchs et al. 2011) to detect any type of cataract in cohorts of different mouse mutant lines. A group size of 19 mice was chosen, because initial statistics showed that under such conditions a difference of 2% opacity between two groups of mice is statistically significant (*p* = 0.05) (Puk et al. 2013a).

Similar to previous reports about an increased radiation sensitivity of *Atm* or *Mrad9* heterozygous mutants for cataracts (Worgul et al. 2002, 2005; Hall et al. 2006; Kleiman et al. 2007), we tested for increased genetic susceptibility in heterozygous *Ercc2* mutants (also known as *Xpd*; Kunze et al. 2015). The *Ercc2* gene product is involved in DNA repair (Fuss and Tainer 2011; Van Houten et al. 2016), and homozygous mutants develop cortical and nuclear cataracts. Moreover, peripheral lymphocytes from heterozygous mice irradiated by 1 Gy (^137^Cs) showed significantly more γH2AX foci than lymphocytes of wild-type mice 6 h after irradiation demonstrating a higher sensitivity of the heterozygotes to ionizing radiation (Kunze et al. 2015). Additionally, epidemiological studies showed that polymorphisms in *XPD* are associated with increased risk of cataracts (Ünal et al. 2007; Padma et al. 2011; Chi et al. 2015).

The whole-body irradiation of the mice allowed us also to follow up other biological end points as survival, cancer development and cytogenetic effects in the bone marrow. This also enabled the comparison of these effects in the same mice without consideration of putative differences in housing conditions, etc.

Importantly, we observed differences between irradiated wild-type and heterozygous mutant mice primarily in the number of chromosomal alterations and telomere length. In contrast, the radiation effects in the survival rate of the mice and in the number of tumors are independent of the genoptype. However, and on the contrary to our expectation, the radiation-induced lens opacities were very subtle.

## Materials and methods

### Mice

We used F1 hybrids of male C3HeB/FeJ and C57BL/6J females as wild types (B6C3F1); as heterozygous mutants we used F1 hybrids from mating male homozygous *Ercc2* mice on C3HeB/FeJ background (Kunze et al. 2015) with wild-type C57BL/6J females (B6RCF1). This breeding schedule was chosen, because the recessive *Ercc2* mutation was on the background of a C3H strain suffering from a recessive retinal degeneration caused by a mutation in the *Pde6b* gene (Pittler and Baehr 1991). To overcome this situation for our analysis of putative retinal changes after irradiation, we crossed homozygous male mutants (homozygous female mutants are sterile) with female C57BL/6J mice resulting in healthy heterozygous *Ercc2* mutants. Importantly, these mice are also heterozygous for the two parental strains (F1 hybrids). Correspondingly, the wild-type controls were crossed in a similar way (wild-type male C3H × wild-type female C57BL/6J). Mice were kept under specific pathogen-free conditions at the Helmholtz Center Munich. The use of animals was in strict accordance with the German Law of Animal Protection and the tenets of the Declaration of Helsinki. The lifetime study was approved by the Government of Upper Bavaria (Az. 55.2-1-54-2532-161-12).

At the age of 10 weeks (± 10 days), groups of 19 mice (wild types and heterozygous mutants, male and female) were whole-body irradiated by doses of 0, 0.063, 0.125 and 0.5 Gy (dose rate 0.063 Gy/min; ^60^Co source in Eldorado 78 teletherapy irradiator, AECL, Canada); the control animals (0 Gy) had the same type of movement and other conditions of exposure, but without dose (sham irradiation).

24 months after irradiation, the experiment was terminated and four mice of each group were taken for organ sampling. To obtain also data during the experiment, additional groups of 16 males and females were irradiated; 4 mice of each group were killed at four different time points (4 and 24 h after irradiation, and 12 and 18 months after irradiation). Organ sampling from mice of this cohort was done for a series of studies which are actually ongoing and will be published later. In the paper here, we describe the results of the cytogenetic study and the telomere length derived from bone marrows of these mice. The different cohorts run between May 2013 and February 2016; each cohort contained a part of the non-irradiated controls to minimize putative seasonal effects.

The mice surviving for 24 months and which were not used for organ sampling underwent detailed pathological examinations (in total: 211 mice). For microscopic histological analysis, all organs (skin, heart, muscle, lung, brain, cerebellum, thymus, spleen, cervical lymph nodes, thyroid, parathyroid, adrenal gland, stomach, intestine, liver, pancreas, kidney, reproductive organs and urinary bladder) were fixed in 4% buffered formalin, embedded in paraffin, sectioned at a thickness of 2 µm and subjected to hematoxylin and eosin staining as described before (Fuchs et al. 2011). In detail, evaluation of inflammation and any reported morphological alterations was done using light microscopy of HE-stained slides of paraffin-embedded standardized sets of organs of all 211 animals as well as of the animals that died before the final killing date. All pathological findings of each animal have been given in a detailed report. For statistical analysis, the diagnoses were summarized in the given headers by present/absent. Regarding inflammation, the following criteria were applied: increased number of mixed infiltrates of granulocytes, lymphocytes and occasional macrophages.

### Cytogenetic analysis

At four different time points (4 and 24 h after irradiation, and 12 and 18 months after irradiation), bone marrow cells were isolated from three femurs of individual mice of each group by flushing out the tissue from the diaphysis of the bones. The isolated cells were suspended in 10% fetal calf serum (Sigma, Taufkirchen, Germany) and MEMα medium (Invitrogen, Karlsruhe, Germany) as reported previously in more detail (Szatmári et al. 2017). The cells were arrested during mitosis by Demecolcine (Sigma) for 1 h at 37 °C and fixed in acetic acid in methanol (1:3 fixative). Cells treated in this way were kept on ice packs for transport. Upon receipt, each sample was divided into 2 × 5 ml aliquots; one aliquot from each sample was further used for telomere length analysis (see below). The remaining 5 ml aliquot samples were prepared for chromosomal analysis. Briefly, each sample was warmed to room temperature and centrifuged at 180×*g* for 10 min; the supernatant was aspirated and the pellet re-suspended in 2 ml of fresh 1:3 fixative. Single-use fine-tip Mini Pastettes (Alpha Laboratories Ltd, Eastleigh, UK) were used to resuspend the cells before transferring a single drop of each sample onto the center of degreased microscope slides. This process of layering cells was repeated until there was a reasonable coverage of cells on each microscope slide. Depending on the sample’s mitotic index, two to four slides were prepared from each sample. Samples were then air dried at room temperature for 24 h prior to staining with 6.7% Giemsa Stain improved R66 solution Gurr^®^ (VWR, Lutterworth, UK) in phosphate buffer (pH 6.8; VWR catalog number 363112P). Slides were again air dried before addition of coverslips secured with Entellan^®^ new rapid mounting media (VWR). Slides were coded and where possible 50-well spread metaphases were analyzed from each sample using a light microscope and 100× objective. For two samples, it was only possible to score 44 and 46 metaphases/sample (male wild type, 4 h, 0 Gy; female mutant, 12 month 0.5 Gy, respectively). Statistical analysis (ANOVA) included chromosomal aberrations based on misrepair of DNA double-strand breaks (i.e., dicentrics, translocations, rings).

### Telomere length determination

Mouse bone marrow cells were suspended in fixative (1 part glacial acetic acid and 3 parts methanol) and shipped on dry ice. For telomere length measurement of these cells, the IQ-(interphase quantitative) FISH method was used. The cells were dropped onto slides and hybridized with PNA telomeric probe. Images were taken using the Carl Zeiss Axioplane 2 microscope and analyzed using the IPLAB software (Digital Scientific). Telomere fluorescence, which corresponds to telomere length, was calculated as described previously (Virmouni et al. 2015).

### Ophthalmic investigations

The transparency of the eye lens was investigated monthly by Scheimpflug imaging (the group size of *n* = 19) allows us to calculate a difference of 2% transparency to be statistically significantly different between the two groups at the level of *p* = 0.05 (Puk et al. 2013a).

Similarly, effects on the retina were investigated in a non-invasive manner every 4 months after irradiation using optical coherence tomography (OCT) essentially as described previously (Puk et al. 2013b). Measurements included counting of the main blood vessels and virtual sections through the retina; the evaluation of the retinal layers and calculation of the retinal thickness were performed by the provided software. The breeding scheme outlined above allowed to investigate radiation effects on the retina, both in wild-type and heterozygous *Ercc2* mutant mice.

### Primary lens epithelial cells

The eyeballs of 10-week-old mice were collected in prewarmed phosphate-buffered saline (PBS; 37 °C). In a Petri dish with warm (37 °C) suspension medium (medium 199 containing Antibiotic–Antimycotic; Gibco, Thermo Fisher Scientific, Waltham, MA, USA) the lenses were prepared, and the lens capsules with attached lens epithelial cells (LECs) were removed and collected in a 1.5 ml Eppendorf tube containing warm, sterile culture medium (medium 199 containing 10% BSA, 5% FBS, Antibiotic–Antimycotic and 100 ng/ml FGF-2). After an overnight incubation at 37 °C, the tubes were centrifuged for 5 min at 2000 rpm (centrifuge EBA 12 with rotor 1412, Hettich, Tuttlingen, Germany). The medium was removed carefully and lens capsules with LECs were suspended in 250 µl Accutase (Gibco, Thermo Fisher Scientific). After 10 min of incubation at 37 °C, the culture medium was added and LECs were suspended by pipetting up and down the medium twice. LECs were spread in 24-well plates and incubated at 37 °C with 5% CO_2_. 5 to 6 days later, the LECs were detached by using Accutase, suspended in culture medium and transferred to µ-Slide 8-well plates (Ibidi, Martinsried, Germany) for irradiation and γH2AX foci assay.

### Lymphocytes from spleen

For the preparation of lymphocytes, spleens of 10-week-old mice were isolated, homogenized carefully and suspended in 2 ml autoMACS running buffer (Miltenyi Biotec, Bergisch Gladbach, Germany). The suspension was centrifuged for 10 min at 200×*g* at 4 °C. The pellet was washed in 15 ml autoMACS running buffer, centrifuged for 10 min at 300×*g* and resolved in autoMACS running buffer (90 µl per 10^7^ cells). For the separation of lymphocytes, CD45 MicroBead technique was used. Magnetic separation was prepared according to the manufacturer’s instructions (Miltenyi Biotec). Comparable to the mice, LECs and lymphocytes were irradiated in the same dose range: LECS by doses of 0, 0.063, 0.125, and 0.5 Gy (dose rate 0.063 Gy/min; ^60^Co source in Eldorado 78) and lymphocytes by 0, 0.050, 0.100 and 0.5 Gy (dose rate 0.45 Gy/min ^137^Cs source HWM D2000). After irradiation, DNA damage was determined using the γH2AX foci assay.

### γH2AX foci assay

Cells (lymphocytes and LECs) were placed in the incubator (37 °C, 5% CO_2_); 1, 4 and 24 h after irradiation, they were fixed in 2% PFA for 15 min. Wells with fixed LECs were rinsed with phosphate-buffered saline (PBS), incubated three times for 5 min with PBS containing 0.15% Triton-X100 and blocked three times for 10 min with blocking solution (PBS containing 1% BSA and 0.15% glycine). LECs were incubated with the primary antibody Phospho-Histone H2A.X (Ser139) (20E3) (NEB, CellSignaling, Frankfurt am Main, Germany) 1:400 in blocking solution overnight at 4 °C. LECs were washed with PBS three times and incubated with an anti-Rabbit IgG (H + L) secondary antibody, Alexa Fluor 488 (Thermo Fisher Scientific) 1:250 in blocking solution for 1 h at room temperature. Afterwards, the cells were washed in PBS three times and incubated with DAPI (D9564, Sigma Aldrich Chemie, Taufkirchen, Germany; dilution 1:10,000) for 15 min, washed with PBS twice, air dried in the dark and covered with mounting medium (Ibidi, Martinsried, Germany). For foci analysis, an automated scanning and analysis system was used (Axioplan 2; Carl Zeiss, Jena, Germany; Metafer4, Metasystems, Altlußheim; Germany).

For lens cells, two biological replicates were prepared independently with a latency of 2 months. Each biological sample was gained by pooling lens cells of 30 mice and processed in three technical replicates. Except for cultures lacking quality control criteria, all data points were included in the regression analysis. For lymphocytes, two mice per strain and sex were analyzed without technical replication. All cultures reached quality criteria.

The γH2AX data were analyzed using the mean number of foci detected per slide. Cells with more than 20 foci were excluded from the analysis. For lens cells, the range of cells per slide was 161–209. For lymphocytes, the range of cells per slide was 267–1014 (except 1 sample with 24 cells: 24 h, mutant, 0.05 Gy).

Linear regression models for the dose response with factors sex, genotype and their interactions with dose were fitted to γH2AX data for each time point and cell type separately. Therefore, differences in variances within the organs could not perturb the calculated effects. Since no sex effect was detected in lens cells, sex as a factor was removed from the lens model. In contrast, for lymphocytes sex and sex–dose interaction were included. Mean effect sizes and the corresponding standard errors were reported. Statistical significance was assigned for *p* < 0.05. All statistical calculations were performed using R (version 3.2.1).

### General statistical modeling

For all models, sex, dose and genotype were used as classification variables. For the body weight data, a linear regression model with fixed and random effects was applied. The above classification variables were included as fixed effects, and to allow individual time courses for each mouse a linear and quadratic term for time as well as an intercept term were included as random effects in the linear mixed model. For the survival analysis, Kaplan–Meier estimates were done, whereas Cox regression analyses were used for the histological data. For the Scheimpflug data, additional analyses for the average value over the last 4 months and over the first 4 months were done by the ANOVA model. These analyses were calculated using the statistical software SAS Version 9.3.

## Results

### General observations

**Fig. 1:**
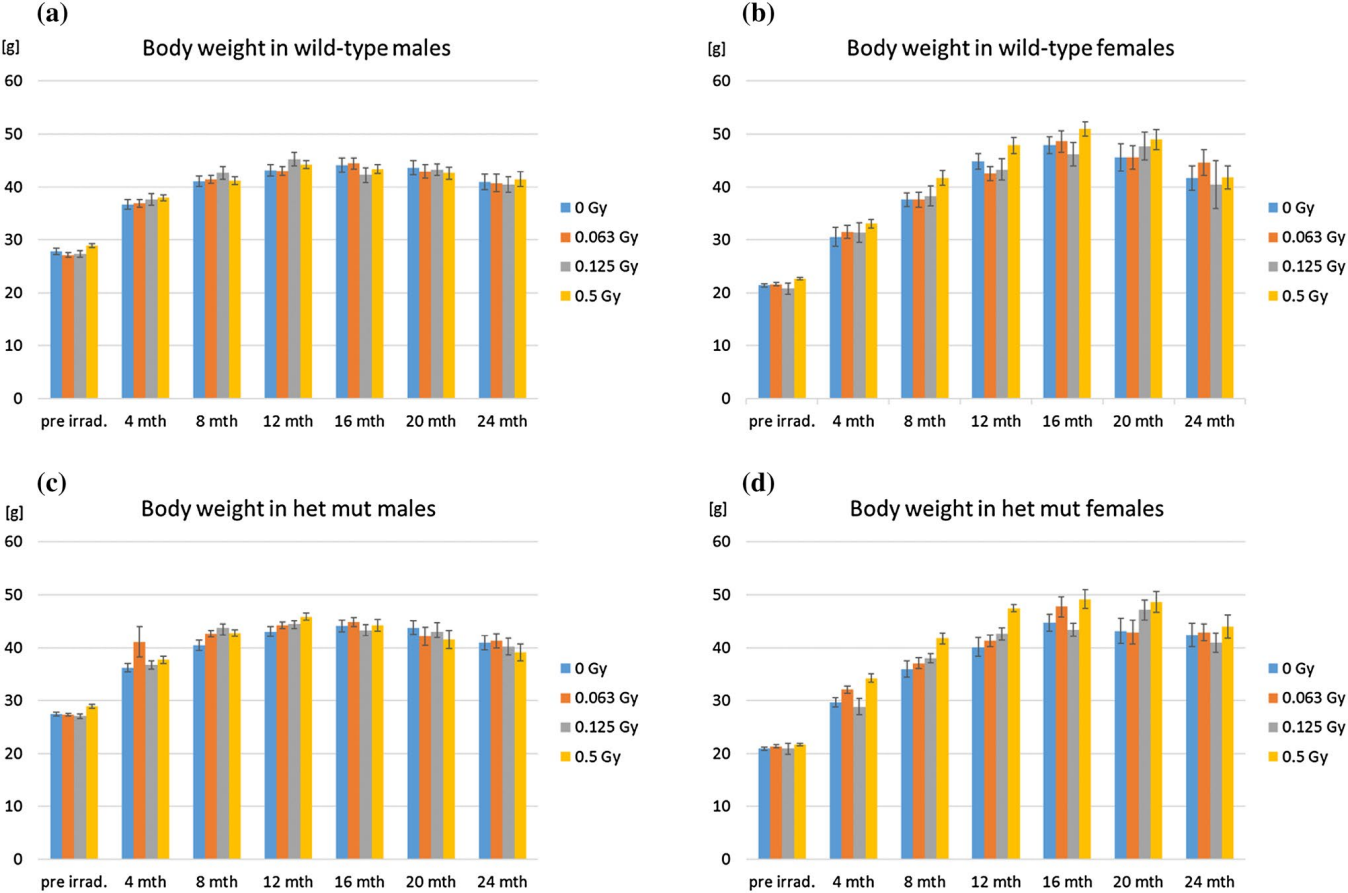
Body weight. Body weight was measured before irradiation (pre irrad.), and then every 4 months post-irradiation. There was no statistically significant difference between wild types and heterozygotes (*p* = 0.426), the development of body weight over the lifespan is shown for **a** wild-type males and **b** females as well as **c** heterozygous mutant (het mut) males and **d** females after different irradiation doses (color coded). Bars represent 10–20 mice, dependent on the survival (see Fig. 2). Error bars represent SEM

The body weight of all mice in the experiments was determined before irradiation, 2 weeks after irradiation and later every 4 months. The results are shown in Fig. 1 indicating an increase in body weight till 1 year after irradiation in males and till 16 months after irradiation in females, followed in all cases by a decline. Among these features, it is obvious that independent of the genotype, females are ~ 6 g (mean) lighter than males during the entire lifetime (*p* < 0.0001). Moreover, independent of sex or genotype, there was a significant effect of radiation dose on body weight over the entire lifespan (*p* < 0.0001). As illustrated in Fig. 1, at the highest dose (0.5 Gy) body weight was increased in both males and females irrespective of genotype; this phenomenon has been observed also in the past without further explanation (Congdon 1987; Babbitt et al. 2001). There was no statistical significant difference between wild-type and heterozygous mutant mice (*p* = 0.426).

**Fig. 2:**
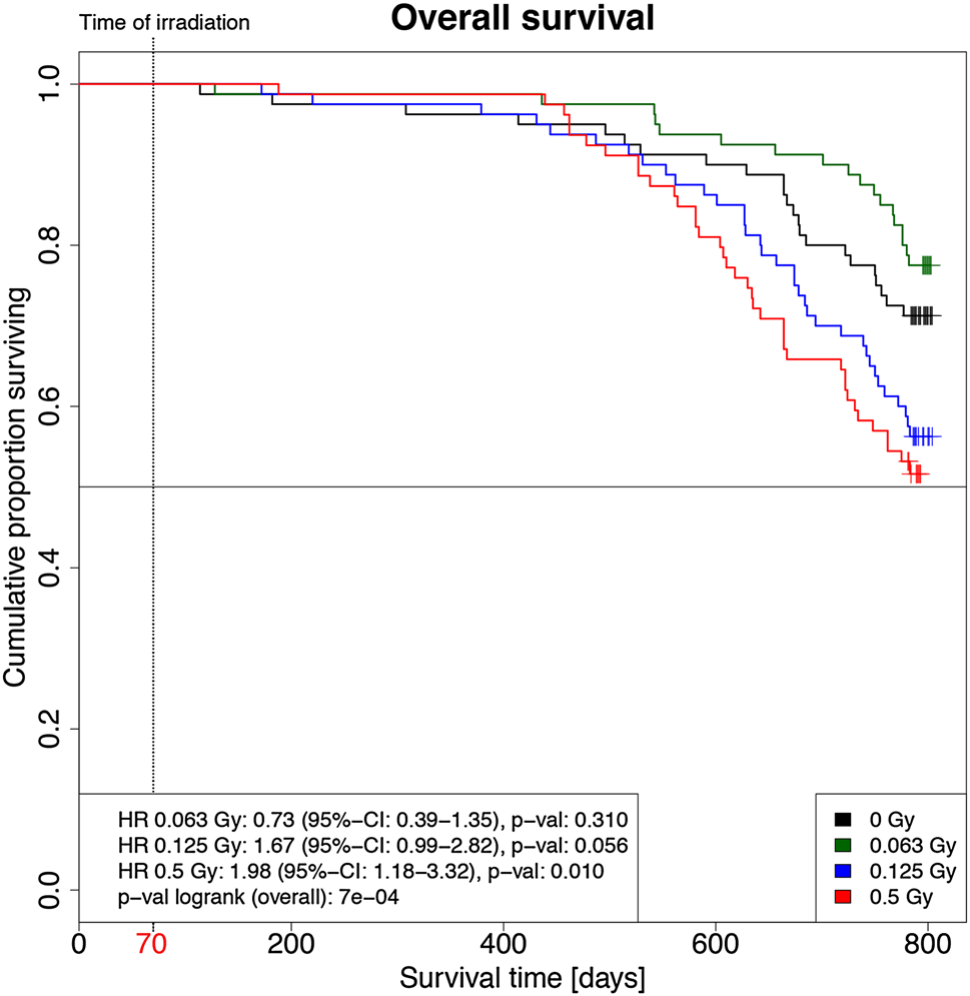
Kaplan–Meier analysis of the overall survival of the mice post-irradiation (p.i.). Kaplan–Meier plots of mice that were irradiated with 0.063 Gy (green), 0.125 Gy (blue) and 0.5 Gy (red) and sham irradiated (black; 0 Gy) are shown. Each group started with 80 mice. The 0.125 and 0.5 Gy groups showed reduced overall survival compared to the sham-irradiated group. Survival of the 0.063 Gy irradiated group was statistically not distinguishable from the sham-irradiated group (*HR* hazard ratio, *CI* confidence interval); mice were irradiated on day 70 (red), while the total lifetime was 800 days

Since the life span of mice varies between 2 and 3 years (Yuan et al. 2011), we noted every dead mouse before the end of the experiment 24 months after irradiation. Figure 2 shows the survival curve of the mice over their lifetime. Radiation was administered at 10 weeks (70 days) of age. One year later, ~ 90% of the mice exposed to radiation survived. This number decreased further after 18 months in the highest dose group to ~ 75%. After 2 years, it dropped down in the highest dose group to ~ 50% with a trend that the female wild-type mice had a higher risk to die earlier than the corresponding males. However, this is statistically not significant (hazard ratio 1.33, CI 95% 0.92–1.93; *p* = 0.131). There is also no statistically significant difference between wild types and mutants (*p* = 0.430). However, the mice of the 0.5 Gy irradiation group showed the highest numbers of premature deaths, independent of sex and genotype; there was a statistically significant dose-dependent increase (*p* = 0.002). In this context, it might be noteworthy that the mice irradiated with the lowest dose (0.063 Gy) seemed to have a higher probability to survive than the non-irradiated mice and the mice irradiated with higher doses. Yet, this effect was statistically not significant.

Detailed pathological examinations were performed in 211 mice 24 months after irradiation, i.e., at an age of ~ 800 days. The pathological findings are summarized in Table 1: remarkably, there was no statistically significant difference between the heterozygous mutants and the wild types for any of the pathologies investigated here. In most of the pathological parameters, there was a statistically significant increase in the highest dose group (ovary tumors, pituitary adenomas, other tumors and inflammation) and in females (ovary tumor, pituitary adenoma and other tumors including mammary carcinomas). On the other side, it is noteworthy that no radiation-induced increase in thyroid adenomas was observed in our cohort of mice, but the risk to suffer from “other tumors” was significantly lower in the lowest dose group compared to the control.

**Table 1:** Pathological findings in mice 24 months after irradiation

	Sex (female vs. male)	0.063 Gy (vs. 0 Gy)	0.125 Gy (vs. 0 Gy)	0.5 Gy (vs. 0 Gy)	Line (heterozygotes vs. wild types)
Ovary tumor	**54.43 (7.49; 397.80)** **< 0.001**	0.28 (0.07; 1.06) 0.060	**4.32 (1.31; 14.27)** **0.016**	**12.83 (4.29; 38.31)** **< 0.001**	1.25 (0.68; 2.27) 0.476
Pituitary adenoma	**9.67 (3.29; 28.38)** **< 0.001**	0.86 (0.34; 2.21) 0.760	**3.84 (1.04; 14.14)** **0.043**	**13.99 (3.94; 49.67)** **< 0.001**	0.94 (0.49; 1.82) 0.854
Thyroid adenoma	1.23 (0.57; 2.65) 0.609	1.06 (0.44; 2.54) 0.899	1.67 (0.52; 5.37) 0.393	1.38 (0.25; 7.61) 0.710	0.78 (0.39; 1.63) 0.534
Other tumors^a^	**2.47 (1.43; 4.26)** **0.001**	*0.34 (0.15; 0.76)* *0.008*	1.93 (0.92; 4.04) 0.083	**2.95 (1.42; 6.13)** **0.004**	0.79 (0.48; 1.31) 0.363
Inflammation	0.652 (0.30; 1.41) 0.277	0.69 (0.24; 1.94) 0.481	1.29 (0.40; 4.22) 0.672	**4.01 (1.29; 12.47)** **0.017**	0.85 (0.41; 1.78) 0.666
Age-related alterations^b^	1.25 (0.83; 1.86) 0.285	0.90 (0.53; 1.52) 0.693	**2.78 (1.58; 4.89)** **< 0.001**	1.69 (0.80; 3.57) 0.173	0.86 (0.59; 1.27) 0.460

Data are given as hazard ratio (with lower and upper 95% confidence interval) and the corresponding *p* value below (without correction for multiple testing); increased hazard risk is given in bold; protective effects are indicated by italics numbers

^a^Other tumors include pheochromocytomas, adenomas of the adrenal gland, insulinomas, mamma carcinomas, fibroadenomas, urothelium carcinoma and other endocrine tumors and discrete other squamous epithelium or adenocarcinomas

^b^Age-related alterations were not associated with any tumor: atrophic testes, cysts of the endometrium, increase in the number of spindle cells in the subcapsular adrenal cortex, calcification of the thalamus, deposits of lipofuscin in the adrenal gland; siderosis in the spleen, etc.

### Chromosomal aberrations and telomere length

**Fig. 3:**
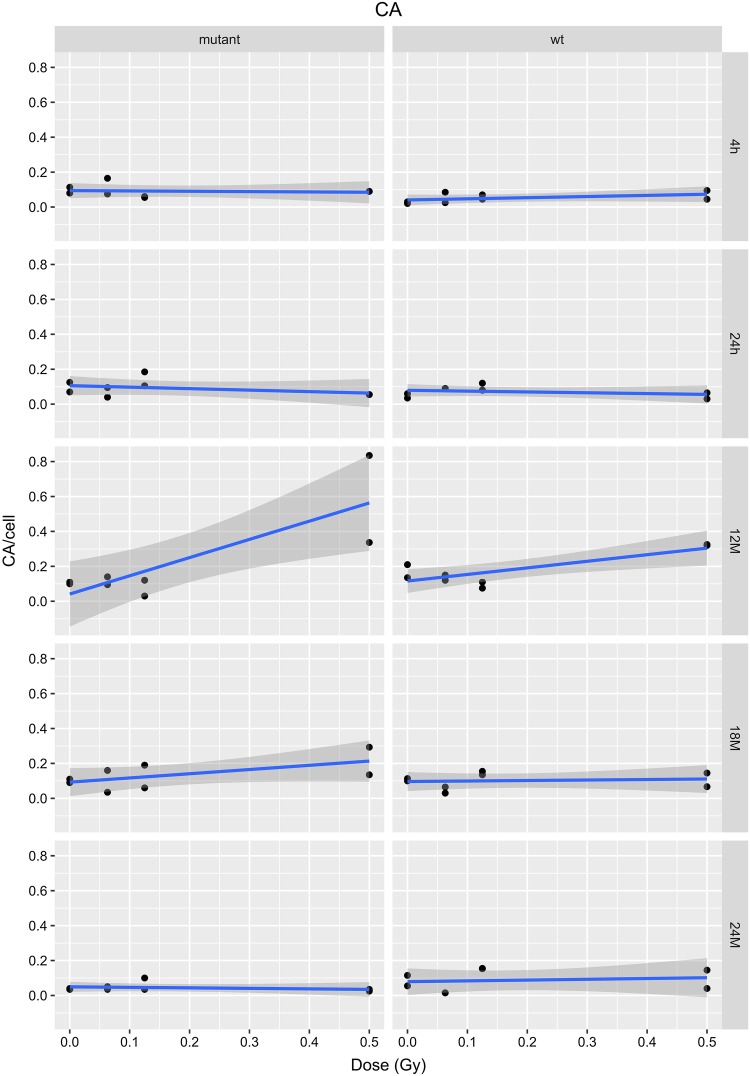
Chromosomal aberrations (CA; dicentric and acentric chromosomes, rings and translocations) in bone marrow cells of irradiated mice. The data presented are based on a linear model for each time point (4 and 24 h, 12, 18 and 24 months after irradiation); the grey areas represent the 95% confidence intervals. The dots represent the pooled mean of three or four male and female samples at each dose given. A dose-dependent increase of chromosomal aberrations is obvious at 12 months after irradiation

Radiation induces chromosomal aberrations such as dicentric and acentric chromosomes, rings and translocations (Romm et al. 2009; Wojcik et al. 2017). In our study, usually 50 metaphases were analyzed from fixed bone marrow cells of femurs of three or four individual mice. The data were pooled resulting in 150 or 200 metaphases per experimental point. There was no statistically significant difference 4 and 24 h after irradiation, but overall we noted a significant effect of the genotype showing a higher risk of chromosomal aberrations in the heterozygous mutants (*p* < 0.001; Fig. 3). The detailed analysis of the interaction of dose dependence and time after irradiation revealed that the differences were mainly due to the data observed 12 months after irradiation (for wild types and mutants: *p* < 0.001). 18 months after irradiation, a statistically significant dose-dependent increase of chromosomal aberrations was observed only for the mutants (*p* = 0.036), but no longer for the wild types (*p* = 0.816; Fig. 3). The total number of chromosomal aberrations is small due to the low dose and dose rate applied to the mice; nevertheless, this result is in line with the observation of a half-life time of 36 months for dicentric chromosomes in human cell culture (Beaton-Green et al. 2016) explaining the decrease of chromosomal aberrations 24 months after irradiation.

**Fig. 4:**
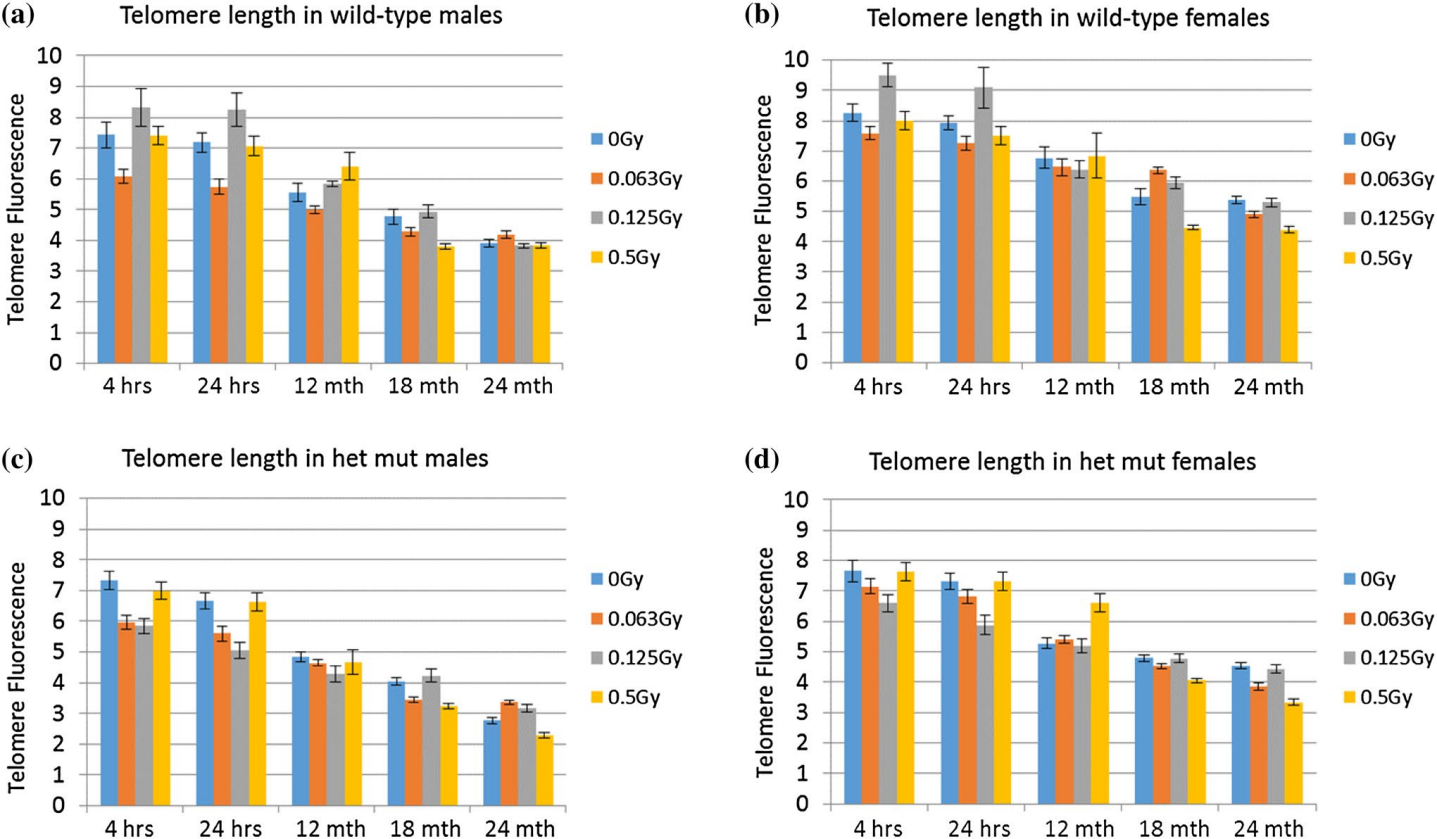
Telomere length. The telomere length (expressed as fluorescence intensity) decreases with increase in age. However, there is no effect associated with the radiation dose (*p* = 0.261). Females have longer telomeres than males (*p* < 0.001) and wild-type mice longer telomeres than heterozygous *Ercc2* mice (*p* < 0.001). A total number of 75 cells were analyzed over a period of three replications. The error bars represent SEM

In the same bone marrow samples as described above, we also measured telomere length. The decrease in telomere length after 2 years is visible in all groups with a more distinct evidence at the dose of 0.5 Gy, but in no case a significant dose effect was shown (*p* = 0.261) (Fig. 4; Table 2). Female samples have longer telomeres compared to male samples (*p* < 0.001), and wild-type samples have longer telomeres compared to mutant samples (*p* < 0.001), which is in good agreement with previous data (Cherif et al. 2003).

**Table 2:** Telomere length in bone marrow cells

	Telomere length (mean)	95% CI
0 Gy	5.87	5.52; 6.23
0.063 Gy	5.43	5.08; 5.78
0.125 Gy	5.87	5.51; 6.22
0.5 Gy	5.68	5.32; 6.03
Females	6.21	5.96; 6.46
Males	5.22	4.97; 5.47
Mutants	5.21	4.96; 5.46
Wild types	6.22	5.97; 6.47

### Ophthalmic examination

**Fig. 5:**
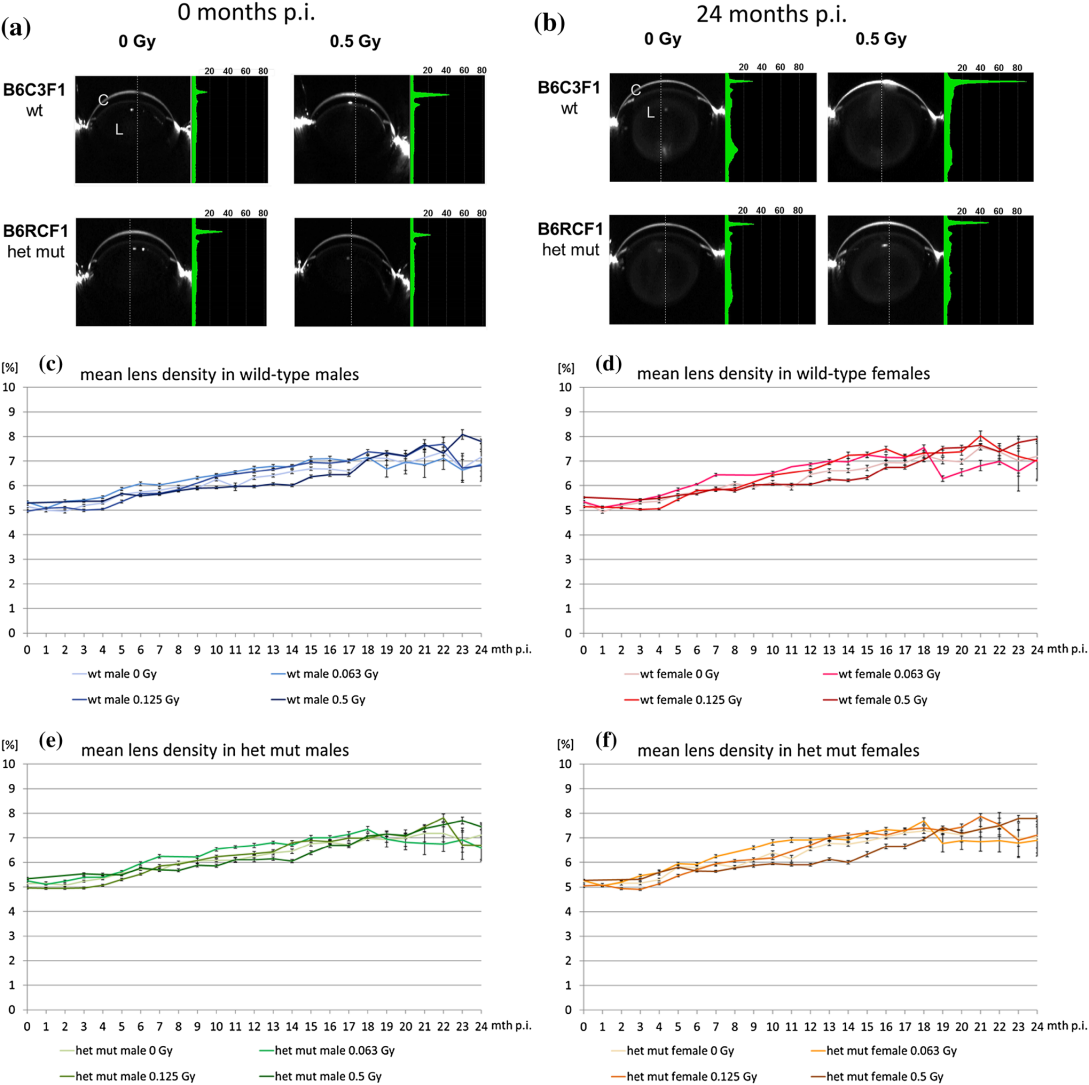
Scheimpflug analysis. The data of the Scheimpflug analysis of lenses are given immediately after irradiation (**a**) or 24 months later (**b**). The figure gives the extreme values (0 or 0.5 Gy) both for B6C3F1 wild-type mice (wt) and B6RCF1 heterozygous mutant mice (females only). The cornea (C) is on the top, and the lens (L) at the center. The bright areas at the left and right side of the eye show hairy skin. The green densitogram represents the percentages of opacity measured at the dotted line. The peaks represent (from top to bottom) the reflections at the cornea and the surface of the lens; 24 months after irradiation, the opacity of the mouse lenses increased slightly. Dose–response curves of mean lens densities of wild-type B6C3F1 males (**c**) and females (**d**) and heterozygous mutant B6RCF1 male (**e**) and female (**f**) mice post-irradiation (p.i.) with 0, 0.063, 0.125 or 0.5 Gy from the time of irradiation to 24 months later are given (color code). The lens opacities have been determined monthly using the Scheimpflug camera. Bars represent standard error of the mean (SEM); *n* ranges between 10 and 20 mice depending on the age

Mice were monthly examined for lens opacities by Scheimpflug imaging up to 24 months after irradiation. As demonstrated in Fig. 5, during these 24 months after irradiation just slight cortical opacities together with faint nuclear opacities developed in all mice indicating an aging effect. The data of the Scheimpflug analysis were plotted against the month of examination after the irradiation as mean lens density (Fig. 5c–f). Since “mean lens density” level gives the data over the entire lens, we used “maximal lens density” for statistical analyses.

Early vs. late effects in the Scheimpflug data were analyzed by ANOVA regarding the values of the maximum lens density over the first 4 months vs. the last 4 months. These summary statistics reduced the heterogeneity in the data compared to single time point analysis. There was no sex difference (*p* = 0.545), and we did not observe an influence of the genotype (*p* = 0.414). The data are given in Table 3, and for illustration of the small differences, the data are given for each dose for females and males separately. In contrast, the effect of the dose is statistically highly significant (*p* < 0.001); however, the dose-dependent increase of the lens opacity is around 1% and, therefore, without clinical relevance. This is also true for the slightly lower increase of the lens opacity over lifetime for the very low dose of 0.063 Gy compared to the other dose groups.

**Table 3:** Maximum lens density in the months 1–4 and 21–24 post-irradiation (p.i.)

	Lens density (%) (months 1–4 p.i.; ± SE)	Lens density (%) (months 21–24 p.i.; ± SE)
Females (Gy)		
0	7.37 ± 0.04	9.52 ± 0.15
0.063	7.38 ± 0.04	8.37 ± 0.14
0.125	7.08 ± 0.04	9.84 ± 0.17
0.5	7.63 ± 0.04	10.37 ± 0.17
Males (Gy)		
0	7.22 ± 0.04	9.35 ± 0.15
0.063	7.31 ± 0.04	8.46 ± 0.14
0.125	6.91 ± 0.04	9.50 ± 0.15
0.5	7.65 ± 0.04	10.52 ± 0.17

To determine any effect on the retina, the retinae of the mice were examined also by OCT (optical coherence tomography) every 4 months. The number of main blood vessels visible in the retinal fundus varied between 8 and 12, which is within the common range. There were no differences between the groups, and no remarkable changes occurred from the first examination at the time of irradiation until 24 months later. No obvious aberrations were detected in the retinal fundus; in particular, there is no neovascularization obvious (data not shown). However, 20 and 24 months after irradiation, we observed significantly thinner retinae in the irradiated mutants only. The quantitative data are given in Supplementary Table 1. To better understand the effect on the retina, we checked whether its thinning is due to a loss of a particular cellular layer or just a shrinking of the entire retina. The first analysis using OCT, however, does not support the loss of a particular layer; this question will be resolved by detailed histology in future experiments and is beyond the scope of this paper. Nevertheless, it should be mentioned here that radiation therapy to the retina using doses under 25 Gy (in fractions of 2 Gy or less) is unlikely to cause significant retinopathy (Archer and Gardiner 1994).

### In vitro data

**Fig. 6:**
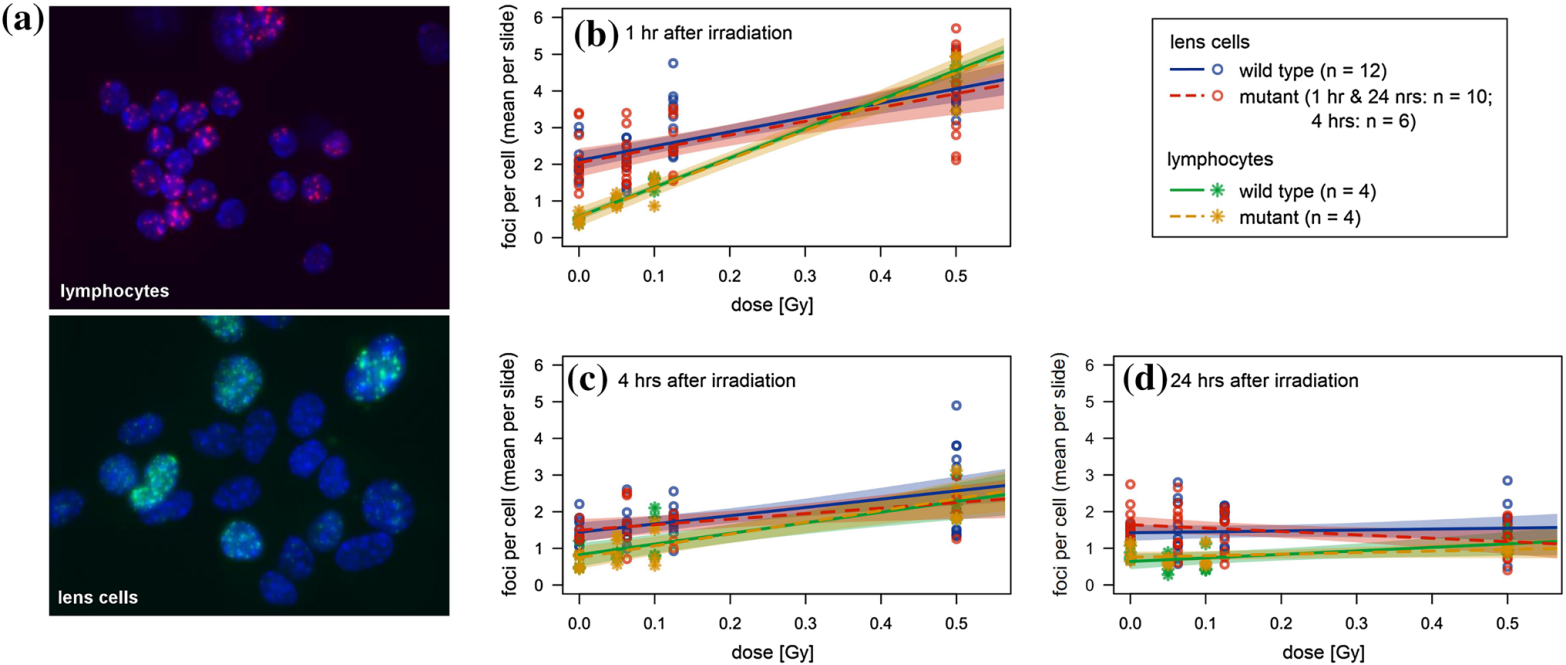
DNA repair in primary lens epithelial cells and in spleen lymphocytes. **a** Primary lens epithelial cells and spleen lymphocytes from wild types and heterozygous *Ercc2* mutants (het) were irradiated by 0.5 Gy, and 1 h later DNA damage was visualized by antibodies against γH2AX (green, Alexa Fluor 488; red, Alexa Fluor 555); cell nuclei were counterstained by DAPI (blue). DNA damage after irradiation was measured by γH2AX response after 1 (**b**), 4 (**c**) and 24 (**d**) h. A significant dose-dependent increase of DNA damage can be observed, which is not detected after 24 h. There is no difference between sex and genotype

To better understand the differences among the various organs analyzed in our lifetime study, we compared the DNA damage after in vitro irradiation of primary lens epithelial cells with that of lymphocytes in the same mouse strains and under the same dose regime used for the lifetime study. Since lens epithelial cells are a quite heterogeneous cell population (Menko 2002; Martinez and de Iongh 2010; Mochizuki and Masai 2014), we enriched growing and dividing cells in the primary cell culture for 5–6 days (see “Materials and methods”). As marker for the DNA damage (i.e., DNA double-strand breaks), we determined the number of γH2AX foci (Löbrich et al. 2010). In lens cells, we did not observe any sex effect: neither basal level (foci at 0 Gy) nor radiation damage (additional foci per Gy) for time points 1, 4, 24 h showed statistically significant differences between male and female mice (data not shown). In contrast, lymphocytes showed significant, but small sex effects after 1 h [radiation damage in male mice was 7.40 ± 0.31 foci per Gy, which is 1.03 ± 0.44 foci per Gy lower than in female mice (*p* = 0.025)] and after 4 h [basal level of male mice was 1.08 ± 0.10 foci, which is 0.58 ± 0.14 foci higher than in female mice (*p* < 0.001)]. Moreover, there were no statistically significant differences between wild types and heterozygous mutants at doses up to 0.5 Gy (Fig. 6b–d; the *p* value for the time points given vary between 0.168 up to 0.927).

It is obvious from Table 4 that the lens cells have a three to four times higher basal DNA damage than the lymphocytes (exact binomial test: *p* = 0.031; after 1 h: *p* < 0.001, *z* test). The damage distribution of the lens cells is more heterogeneous compared to the lymphocytes (Fig. 6a). To cover the depth of a single nucleus, the nuclei were screened in 11 focus plains during the automatic foci analysis. The same procedure and classifier were used for both lymphocytes and LECs. The heterogeneity of the LECs make a quantitative comparison to lymphocytes difficult. However, it is obvious that LECs showed a different DNA-damage response than lymphocytes. This might be due to differences in cell cycle (non-stimulated lymphocytes are in G_0_-phase, and the lens cells might be in different stages of their cell cycle) and/or culture conditions (lymphocytes are cultured in suspension, whereas lens cells grow on plates).

**Table 4:** γH2AX foci as a function of dose

	Basal level at 0 Gy (foci per cell)				Radiosensitivity (foci per cell per Gy)			
	Wild type		Mutant		Wild type		Mutant	
	Mean	SEM	Mean	SEM	Mean	SEM	Mean	SEM
Lens cells (h)								
1 h	2.10	0.15	2.04	0.16	3.91	0.58	3.76	0.63
4 h	1.44	0.12	1.50	0.18	2.25	0.48	1.51	0.68
24 h	1.42	0.11	1.64	0.12	0.24	0.41	− 0.92	0.45
Lymphocytes (h)								
1 h	0.59	0.10	0.58	0.10	8.49	0.39	8.38	0.39
4 h	0.54	0.13	0.46	0.13	2.74	0.50	3.05	0.50
24 h	0.69	0.11	0.80	0.11	0.90	0.42	0.36	0.42

Mean values and corresponding standard errors of the mean (SEM) were estimated in a multiple linear regression model with dose, strain and dose–strain interaction as factors (lymphocytes: *n* = 4 samples per dose point; wild type: *n* = 12; mutant: *n* = 10 at 1 h and 24 h, *n* = 6 at 4 h)

Remarkably, the radiation damage (calculated as foci per cell) is always higher in lymphocytes (exact binomial test: *p* = 0.031)—the increase in the number of foci after 1 h (when foci development reaches its top) is more than two times higher than in lens cells (Table 4; Fig. 6b, *p* < 0.001, *z* test). Taken together, it indicates that lens cells are not as sensitive to radiation as lymphocytes. Also in previously reported comparisons between irradiated lens epithelial cells and lymphocytes, a significant difference in DNA damage is detected at doses higher than 1 Gy (Bannik et al. 2013). In contrast to our results, cells of lens explants showed a higher or almost equal number of γH2AX foci compared to lymphocytes of the same mice (1 or 3 h after irradiation) in whole-body-irradiated mice (0.02 or 0.1 Gy; Markiewicz et al. 2015).

## Discussion

In this study, we asked the question for cataract formation at low doses of ionizing radiation (0.063 and 0.125 Gy) 2 years after irradiation (i.e., the lifetime of a mouse) using the dose of 0.5 Gy as positive control according to a series of experiments performed by Kleiman et al. (2007). As additional control for radiation effects outside the eye, we used several biological end points in the same animals like body weight and survival time, the formation of cancer in different organs and cytogenetic alterations including effects on telomere length in bone marrow cells.

Irradiation with 0.5 Gy shortens the lifespan significantly, whereas the lower doses are without a significant effect. Interestingly, the lowest dose of radiation leads to an apparent, but statistically not significant increase of the lifespan. The reduced lifespan after irradiation with 0.5 Gy cannot be explained by shortened telomeres as we did not observe any significant changes in bone marrow cells dependent on the radiation dose. Generally, our observation of longer telomeres in female mice compared to male mice is in good agreement with the literature (Cherif et al. 2003; Gardner et al. 2014). Moreover, the shorter telomeres in the mutants are in line with previous studies: many DNA repair defective mouse models show abnormalities in telomere length (Slijepcevic 2006).

We observed cytogenetic aberrations (particularly dicentric chromosomes) mainly 12 months after irradiation as consequences of misrepaired double-strand breaks. Because non-symmetric cytogenetic aberrations as dicentric chromosomes show a loss of 50% per cell division, it is obvious that with increasing time since the irradiation the number of such radiation-induced cytogenetic defects decreases. The time, which we observe, is in the same order of magnitude as calculated for such defects in cell culture (Beaton-Green et al. 2016). Moreover, it is obvious that there are radiation-induced tumors, at least in some tissues (Table 1).

However; the most striking result of our lifetime study in mice reported here is the observation that under our experimental conditions, a dose of 0.5 Gy led to an increase of lens opacities just by 1% within a lifespan of 2 years. This is not only unexpected because of the lifespan and tumour effects discussed above, but also in contrast to many previous reports (Worgul et al. 2002, 2005; Hall et al. 2006; Kleiman et al. 2007). Interestingly, these reports demonstrated the formation of cataracts after irradiation with 0.5 Gy at the age of 4 weeks, and earlier papers observed cataracts even at doses of ~ 0.33 Gy (grade 2, after 20 months), if the mice were irradiated at 8–14 weeks of age (Upton et al. 1956).

In all of the previously published papers, cataracts have been observed via slit lamp and graded according to Merriam and Focht (1962). The Merriam-and-Focht scoring system was developed using acute high-dose irradiation (40–50 Gy) of the central part of the lens in whole-body-shielded rabbits or rats. This high-dose irradiation leads to a marked depression of the mitotic activity in the central part of the anterior lens epithelium, the germinative zone, for 6 months. The first sign of cataracts (1+) appeared as central posterior subcapsular vacuoles and dots, followed by an increase in the posterior cortical and beginning of the central anterior opacity (2+). Stage 3+ is characterized by extension of changes in both the anterior and posterior lens cortex; eventually, the lens is completely opaque (4+). The initial phase of this process, the formation of posterior-subcapsular vacuoles and dots, is believed to be caused by epithelial cells escaping the differentiation to lens fiber cells and migrating along the lens capsule to its posterior part (Wiley et al. 2011). According to the classical scoring scheme (introduced by Merriam and Focht 1962), vision-impairing cataracts have been classified by a score of 2.0 or higher (Kleiman et al. 2007).

In clinical ophthalmology, a similar lens opacity classification system was developed with LOCS III (Chylack et al. 1993) as one of the most frequently used ones. Again, an LOCS score > 2 is defined as having cataract and compatible with visual impairment (Tang et al. 2015).

However, during recent years, the Scheimpflug method for quantification of lens opacity was developed. Compared to slit lamp retroillumination, the Scheimpflug system allows objective quantification of posterior capsule opacification much easier because the images are free of flash reflections (Jain and Grewal 2009). Using the Scheimpflug technique, Jeon and Kim (2011) observed 26% posterior-subcapsular cataracts among highly myopic patients who underwent cataract surgery, and Abe et al. (2013) reported also posterior-subcapsular opacities after radiation exposure.

To the best of our knowledge, Pei et al. (2008) is the only paper comparing LOCS III grading, Scheimpflug data and visual acuity: first of all, the lens density and LOCS grading for nuclear opacities corresponded almost perfectly (correlation coefficient is 0.965). According to this system, a lens density of 10.5% corresponds to the LOCS-III score of 1.9 and a visual acuity of 0.01 (in the logMAR system, which is very close to 1 in the decimal system).

Similar to clinical ophthalmology, the Scheimpflug system was successfully introduced to analyze lens opacities also in the mouse. A sample size of ~ 17 mice per group allows a difference of 2% of lens density to be calculated, being statistically significant (Puk et al. 2013a), which was shown to be the aging effect on lens transparency in the mouse lens (Fig. 5 this manuscript, but also Puk et al. 2013a). Over the lifetime of the mouse, the radiation effect adds ~ 1% resulting in a maximum lens density in females of 10.4% and males of 10.5%— both values correspond to an LOCS-III core of less than 2 and are, therefore, not considered to be vision impairing (Pei et al. 2008).

One might argue that light scattering due to random fluctuations of lens densities might play a major role for vision impairment. However, the data by Pei et al. (2008) indicate that visual acuity corresponds more strongly with the lens density value than with an LOCS-III score indicating again that an objective method would seem to be more valid and sensitive in estimating lens opacity and visual impairment (Pei et al. 2008).

Since it is well known that various strains of mice show different sensitivity to radiation (Roderick 1963), it might be argued that the strains of mice used in our experiments reported here are rather resistant against ionizing radiation. However, this seems unlikely, as we observed radiation-induced cancers and chromosomal aberrations in other organs from these mice despite not seeing cataract formation. Also, the statistically significant shorter survival time after 0.5 Gy indicates that there was a significant effect of radiation on the general health of these mice. Thus, we conclude that in these mice irradiated at young adult ages, the lens is not as radiation sensitive as other organs, and as previously considered. In future experiments, we will address these questions by using different mouse strains, higher doses and/or dose rates and correlate the lens density in the mouse also to visual acuity.

## Electronic supplementary material

Below is the link to the electronic supplementary material.

Supplementary material 1 (PDF 133 KB)
