# Supplementary material for: Lifetime study in mice after acute low-dose ionizing radiation: a multifactorial study with special focus on cataract risk
Source: Radiat Environ Biophys. 2018 Jan 11;57(2):99–113. doi: 10.1007/s00411-017-0728-z (PMC5902533; doi:10.1007/s00411-017-0728-z)
Supplement: Supplementary file 1 — Supplementary material 1 (PDF 133 KB) [file 411_2017_728_MOESM1_ESM.pdf]

Supplementary Table 1: Retinal thickness

		0 months p.i.	20 months p.i.	24 months p.i.
male wt	0 Gy	253.19 ± 1.02	251.79 ± 2.02	247.67 ± 1.88
male wt	0.5 Gy	256.03 ± 0.82	250.50 ± 1.22	251.80 ± 1.44
female wt	0 Gy	253.81 ± 0.86	248.64 ± 0.95	242.83 ± 1.11
female wt	0.5 Gy	258.89 ± 1.13	248.80 ± 1.38	249.12 ± 1.77
male het mut	0 Gy	254.06 ± 0.80	251.38 ± 1.07	252.50 ± 2.15
male het mut	0.5 Gy	253.75 ± 1.29	246.45 ± 1.53 p=0.013	246.95 ± 1.59 p=0.050
female het mut	0 Gy	251.94 ± 0.94	247.50 ± 1.25	247.30 ± 1.45
female het mut	0.5 Gy	254.65 ± 1.13	243.60 ± 0.96 p=0.021	239.32 ± 1.01 p<0.001

Mean retinal thickness in µm ± SEM. The p values are given for significant differences in retinal thickness between control (0 Gy) and 0.5 Gy irradiated heterozygous mutant male and female mice 20 and 24 months post irradiation.
